# The Effects of Aspirin Intervention on Inflammation-Associated Lingual Bacteria: A Pilot Study from a Randomized Clinical Trial

**DOI:** 10.3390/microorganisms12081609

**Published:** 2024-08-07

**Authors:** Guillaume C. Onyeaghala, Shweta Sharma, Mosunmoluwa Oyenuga, Christopher M. Staley, Ginger L. Milne, Ryan T. Demmer, Aasma Shaukat, Bharat Thyagarajan, Robert J. Straka, Timothy R. Church, Anna E. Prizment

**Affiliations:** 1 Division of Nephrology, Hennepin Healthcare, University of Minnesota, Minneapolis, MN 55415, USA; onyea005@umn.edu; 2 Division of Epidemiology and Community Health, University of Minnesota, Minneapolis, MN 55455, USA; sharm842@umn.edu (S.S.); thya0003@umn.edu (B.T.); 3 Department of Internal Medicine, SSM Health St. Mary’s Hospital—St. Louis, St. Louis, MO 63117, USA; oyenugamosun@gmail.com; 4 Department of Surgery, Medical School, University of Minnesota, Minneapolis, MN 55455, USA; cmstaley@umn.edu; 5 Masonic Cancer Center, University of Minnesota, Minneapolis, MN 55455, USA; trc@umn.edu; 6 Department of Medicine, Vanderbilt School of Medicine, Nashville, TN 37232, USA; ginger.milne@vumc.org; 7 Mayo Clinic College of Medicine & Sciences, Rochester, MN 55905, USA; demmer.ryan@mayo.edu; 8 Department of Population Health, New York University Grossman School of Medicine, New York University, New York, NY 10016, USA; shaukat@umn.edu; 9 Department of Laboratory Medicine & Pathology, Medical School, University of Minnesota, Minneapolis, MN 55455, USA; 10 Department of Experimental and Clinical Pharmacology, College of Pharmacy, University of Minnesota, Minneapolis, MN 55455, USA; strak001@umn.edu; 11 Division of Environmental Health Sciences, School of Public Health, University of Minnesota, Saint Paul, MN 55108, USA

**Keywords:** oral microbiome, periodontitis, colorectal cancer, aspirin

## Abstract

Several bacterial taxa enriched in inflammatory bowel diseases and colorectal cancer (CRC) are found in the oral cavity. We conducted a pilot study nested within a six-week aspirin intervention in a randomized placebo-controlled trial to test their response to aspirin intervention. Fifty healthy subjects, 50–75 years old, were randomized to receive 325 mg aspirin (n = 30) or placebo (n = 20) orally once daily for six weeks. Oral tongue swabs were collected at baseline and week six. We estimated the association between aspirin use and the temporal changes in the relative abundance of pre-specified genus level taxa from pre- to post-treatment. The temporal change in relative abundance differed for eight genus level taxa between the aspirin and placebo groups. In the aspirin group, there were significant increases in the relative abundances of *Neisseria*, *Streptococcus*, *Actinomyces*, and *Rothia* and significant decreases in *Prevotella*, *Veillonella*, *Fusobacterium*, and *Porphyromonas* relative to placebo. The log ratio of *Neisseria* to *Fusobacterium* declined more in the aspirin group than placebo, signaling a potential marker associated with aspirin intervention. These preliminary findings should be validated using metagenomic sequencing and may guide future studies on the role of aspirin on taxa in various oral ecological niches.

## 1. Introduction

Although the overall rates of colorectal cancer (CRC) incidence and mortality have decreased during the past 20 years [1], CRC remains a leading cause of cancer-related death in the United States. The progression of adenomatous polyps (adenomas) in the colon into invasive and metastatic cancerous tumors (carcinomas) is preventable, mainly if adenomas are detected through screening and removed early. The five-year survival rate in CRC survivors is 90% if the diagnosis occurs while the disease is still localized [2]. However, the five-year survival rate for CRC sharply declines to under 15% if invasion or metastasis are detected at diagnosis [1]. This disparity in survival rate highlights the need for understanding mechanisms linked to increased CRC risk.

Many oral pathogens linked to chronic inflammation thrive under conditions associated with dysbiosis: an imbalance in the human microbiome linked to a disease where the ecological balance of the oral cavity is disrupted [3,4,5,6,7,8,9,10]. In turn, this inflammation supports further shifts towards dysbiotic subgingival plaque composition, resulting in the development of gingivitis and periodontitis [7,8,11,12]. Pathogens and pathobionts present in gingivitis and periodontitis can then disseminate into the gut via swallowing, bacteremia, or periodontal pockets following tooth loss [5,9,13,14,15]. Recent studies have linked oral dysbiosis and periodontitis to colorectal cancer (CRC) risk [7,8,14]. Although research on a direct association between the oral microbiome and CRC risk has been limited, several potential mechanisms have been proposed that may explain how oral bacteria can contribute to cancer risk. For example, oral bacteria can convert ethanol to acetaldehyde or nitrosamine to nitrosodiethylamine (NDEA), both recognized carcinogenic compounds [16,17,18,19]. Subsequently, those carcinogens may travel along the gastrointestinal (GI) tract and cause local effects such as the stimulation of DNA methyltransferase or 5’-C-phosphate-G-3 (CpG) island methylation in the colon [20,21].

Another critical pathway through which the oral microbiome may be associated with CRC development is promoting local and systemic inflammatory responses since inflammatory factors are a well-documented risk factor for CRC [22,23]. Certain bacteria found in the oral microbiome have also been detected in colorectal tumor tissue and have been shown to promote inflammation of the intestinal epithelium [24,25]. In addition, these taxa are also involved in chronic inflammatory disorders of the intestine, such as inflammatory bowel diseases (IBDs) [26,27] like Crohn’s disease (CD) [26,27] and ulcerative colitis (UC), [28,29,30] when they are located in the gut microbiome. In particular, *Fusobacterium* and *Porphyromonas* are oral taxa that work synergistically and possess several virulence factors that promote bacterial survival and CRC development [13,24,25,31].

Given the role of inflammation in CRC development, the potential use of aspirin (acetylsalicylic acid) and other nonsteroidal anti-inflammatory drugs (NSAIDs) as chemopreventive agents is an active area in CRC research [32,33,34]. In particular, aspirin irreversibly inhibits cyclooxygenase enzyme (COX)-2 [35], and aspirin-modified COX-2 produces lipoxins, most of which are anti-inflammatory [36]. Aspirin may also exert its anti-inflammatory effect through COX-independent mechanisms, including direct effects on cytokines and transcription factors, and inhibition of oxidative DNA damage. Based on the role of the oral microbiome in promoting local and systemic inflammatory responses, as well as the presence of pro-inflammatory oral taxa in CRC cases, we hypothesized that aspirin could act as a CRC chemopreventive agent either by indirectly counteracting the virulence factors of inflammation-associated taxa [37] or direct inhibitory effects of inflammation-associated taxa such as *Fusobacterium* [38,39]. To test if either of these potential mechanisms may play a role, we conducted this pilot study on the association between aspirin administration and the oral bacterial community within a clinical trial of 50 healthy participants within a pilot double-blind, randomized, placebo-controlled trial.

## 2. Materials and Methods

### 2.1. Parent Study Design

Our study was conducted within the parent study, “Effect of Aspirin on the Gut Microbiome (ASMIC)” [40]. ASMIC was a randomized clinical trial that aimed to determine whether treatment with aspirin results in a favorable shift in the composition of the gut microbiome. As described in the parent study, the trial was registered at www.clinicaltrials.gov (accessed on 24 July 2024, NCT02761486), approved by the Institutional Review Board at the University of Minnesota, and all participants provided written informed consent [40]. The study conformed to the CONSORT reporting guidelines [41].

**Figure 1:**
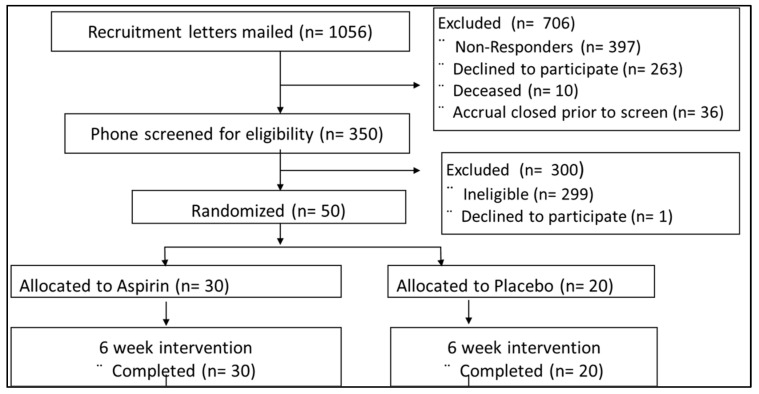
ASMIC trial and intervention CONSORT diagram.

The ASMIC study is a randomized, placebo-controlled, double-blind study that recruited 50 healthy subjects, 50–75 years old, from the PRospective Evaluation of SEPTin-9 (PRESEPT) cohort living in the greater Twin Cities area of Minnesota, USA. A total of 1056 individuals were screened, but of the 350 participants who were willing to participate, 50 met our eligibility criteria (Figure 1). As we previously described in our parent study, the exclusion criteria for this study included: use of any antibiotic prescription in the last 3 months; use of any NSAIDs > 2 times a week in the last 3 months; use of antiplatelet or anticoagulant medication, medications for diabetes or hypertension within the past 30 days; gastrointestinal (GI) cancer or any serious GI condition or surgery within 6 months; any serious active medical (cancer, CVD) or psychiatric illness; BMI ≥ 40 or ≤17 kg/m^2^; unexplained change in weight of >4.5 kg within the past 6 months; or major changes in eating habits within the past 3 months [40]. As this was a pilot study nested within a pre-existing trial, we were unable to conduct oral examinations as they were outside the scope of the parent study.

Upon confirmation of eligibility, the subjects were asked to refrain from consuming any other NSAIDs and over-the-counter medications containing NSAIDs, and from having significant changes in their diet for the duration of the study. Participants were randomized into an aspirin (n = 30) and placebo arm (n = 20) using a block randomization scheme (n = 5 per block) to ensure a 3:2 balance.

For the intervention arm of the study, participants received a 325 mg aspirin capsule once a day for six weeks, and the six-week intervention period was followed by six weeks of washout. In the placebo arm of the study, participants received a 325 mg lactose pill as a placebo once a day for six weeks, and the six-week intervention period was followed by six weeks of washout (Figure 1). With the exception of the study statistician and study pharmacist, all study participants and all study staff were blinded to the treatment given. The study statistician and pharmacist had a blind code generated to identify the content of the capsule.

### 2.2. Data Collection

At baseline, every patient scheduled a clinic visit to obtain informed consent and collect demographic information (Visit 1, Week 0). During this initial visit, participants finished a brief medical and dietary history. Participants also attended a clinic visit after the six-week intervention period (Visit 2).

### 2.3. Sample Collection

The blood, urine, and oral samples were collected by trained study staff during the two clinic visits at week 0 (Collection 1 at Visit 1, before the intervention) and week 6 (Collection 2 at Visit 2, after the intervention). Urine samples were collected in the parent study to assess the effectiveness of aspirin treatment using the urinary metabolite of prostaglandin E2, PGE-M, adjusted for creatinine levels. Urinary concentrations of (PGE-M), were measured in the Eicosanoid Core Laboratory at Vanderbilt University Medical Center, and urinary creatinine levels were measured using a test kit from Enzo Life Sciences. The urinary metabolite levels in each sample were normalized using the urinary creatinine level of the sample and expressed in ng/mg creatinine [42,43]. The oral samples were self-collected by participants using MoBio Catch-All tongue swabs after instructions from the study staff. The swab samples were then frozen at −20 °C after collection. After collection, blood and urine samples were aliquoted and then frozen at −20 °C. Staff transferred all samples to −80 °C within 24 h of sample collection.

### 2.4. DNA Extraction

All bacterial DNA extraction, sequencing, and amplification were conducted at the University of Minnesota Genomics Center (UMGC). To characterize the oral microbiome of the study participants, bacterial DNA was extracted from tongue swab samples using the Powersoil DNA extraction kits (QIAGEN, Hilden, Germany) according to the manufacturer’s instructions [44]. Quality control on the final DNA product was performed using a spectrophotometer reading. DNA purity was assessed using the Qubit dsDNA High-Sensitivity Kit to measure the A260/280 DNA Yield test [45,46,47].

### 2.5. DNA Amplification and Sequencing

The V4 variable region of the 16S rRNA gene from each DNA sample was amplified and sequenced using validated DNA probes and the Illumina MiSeq Personal Sequencing platform (2 × 300 Paired-end) using the 515F-806R primer set, generating 4,656,874 reads (median reads per sample = 49,604) [48,49]. The UMGC facility used sterile water controls for each batch during the DNA amplification steps. In addition, two wells on each 96-well sample plate were reserved for positive and negative controls. Quality control on the final DNA product was performed using the Qubit dsDNA High-Sensitivity Kit for DNA yield to measure the A260/280 to test for DNA purity [45]. After the analysis, the sequenced genetic data was archived in the Sequence Read Archive at the National Center for Biotechnology Information under BioProject accession number SRP127801 [50].

### 2.6. Bioinformatics Analysis

DADA2 bioinformatics software (version 1.6.0) was used to process and analyze the sequence data, trimming forward and reverse reads to 200 nt to remove low-quality reads and merge the results. High-quality sequences were aligned against the SILVA database version 132. After our taxonomy assignment, all subsequent analyses were restricted to bacterial groups representing at least 1% of total reads classifiable at each taxonomic level.

### 2.7. Statistical Analysis

We tested for differences in the demographic characteristics of the ASMIC participants using *t*-tests for continuous variables and chi-square tests for categorical variables. In addition, we evaluated pre-post treatment change in PGE-M levels, adjusting for baseline PGE levels, to check for compliance with aspirin intake, as PGE-M production is suppressed by aspirin intake and served as a marker of treatment compliance for our study.

The bioinformatics analysis described in the “Taxonomy Assignment” section generated features (i.e., bacterial taxa) for the oral microbiome samples. We estimate α-diversity using the Shannon index as the primary metric [51,52,53]. The differences in alpha diversity measures between the aspirin and placebo groups from baseline and post-intervention (after six weeks) were assessed using linear mixed-effect regression models. These models were further adjusted for age, gender, and BMI.

We assessed *β*-diversity by calculating the Bray-Curtis distance measure on the rarefied and log-transformed abundance data. Then, we summarized the Bray-Curtis values for the aspirin and placebo groups using principal coordinates analysis (PCoA). We compared the microbial composition of the samples between baseline and post-intervention using the analysis of similarities (ANOSIM) function in Mothur (ver. 1.35.1) to test the statistical significance of the difference in *β*-diversity between two or more groups of sampling units: the aspirin group at baseline, placebo group at baseline, aspirin group post-intervention, and placebo group post-intervention. We also assessed *β*-diversity using permutational multivariate analysis of variance (PERMANOVA), as implemented by the Adonis function in the vegan package. PERMANOVA was used to test the differences in *β*-diversity measures between the aspirin and placebo groups post-intervention (after six weeks).

### 2.8. Primary Analyses

Using intention-to-treat (ITT), the outcomes in the main analysis were (1) the differences in the microbial community composition and abundance (*α*- and *β*-diversity) at baseline and after the six-week treatment, and (2) the change in the log-ratio of a priori chosen pro-inflammatory and commensal oral bacterial taxa. Because this was a pilot study, and hence of small size, and because we expected a modest effect, we decided to focus on a small number of individual lingual bacterial taxa identified in previous studies that were either (a) enriched in oral dysbiosis and CRC, or (b) had biological functions relevant to oral health or inflammation in the oral ecological niche [5,7,24,54]. This resulted in the selection of 14 taxa at the genus level, which were analyzed as described below.

To account for the compositional nature of microbiome data, we used a multinomial regression analysis approach based on the songbird package in QIIME2 [55] to identify differentially abundant microbes in the aspirin and placebo groups based on our pre-specified taxa. Each taxon was assigned a differential by comparing the logarithm of the fold change in the relative abundance of all taxa between the aspirin and placebo arm to generate differentials. All taxa were analyzed simultaneously using multinomial regression to generate differentials. From the output of our multinomial regression, we ranked the beta estimates for the differentials in each taxon between arms and found that the genus *Fusobacterium* changed more in the aspirin vs. the placebo group (i.e., ranked higher in the aspirin group compared to the placebo group).

After identifying *Fusobacterium* as a “reference frame” (i.e., the denominator value for log ratios in the analysis, which serves to control for the fact that biospecimen mass is not standardized during collection), we computed the log ratio of *Fusobacterium* to our taxa of interest associated with oral health. Finally, to compare the change over time in the log ratio for each taxon across arms, we used a linear mixed effect regression model in which log ratio values at week 0 and week 6 were entered as repeated values. Significant balances for the change over time in the aspirin vs. placebo group were determined with a *p*-value cutoff of 0.05.

### 2.9. Differential Abundance Analysis

In addition to the primary analyses, we ran a differential abundance analysis using the DESeq2 package to simultaneously test multiple bacterial taxa for the comparison of post-intervention oral samples between the aspirin and placebo treatment arm, after controlling for the expected rate of false positives using a False Discovery Rate Adjustment [56]. 

The analyses were conducted using the DADA2, EdgeR, vegan, phyloseq, and DESeq2 packages in the R Statistical Analysis software package, Version 3.4 (CRAN) (2-sided tests, α = 0.05), and the songbird and QURRO packages in QIIME2.

## 3. Results

### 3.1. Parent Study Cohort

The study cohort included 50 participants who were randomized to the aspirin group (n = 30) and placebo group (n = 20). Participant demographics were balanced for age (mean age in the aspirin group = 62.2 years, mean age in the placebo group = 61.2, *p*-value for difference = 0.56), BMI (mean BMI in the aspirin group = 27.3 kg/m^2^, mean BMI in the placebo group = 28.2 kg/m^2^, *p*-value for difference = 0.49), and for baseline PGE-M levels (mean PGE-M levels in the aspirin group = 11.82 ng/mg creatinine, mean PGE-M levels in the placebo group = 12.94 ng/mg creatinine, *p*-value for the difference = 0.74) (Table 1). After six weeks of intervention, 47 participants (94%) had at least 90% capsule compliance, and changes in urinary PGE-M between the aspirin and placebo groups [−6.17 (95% CI: −9.16; −3.18) mg/dL, *p* < 0.01] indicated high treatment compliance, as well (Table 1).

### 3.2. Analyses of α- and β-Diversity

When the analysis was conducted in each arm, we found that α-diversity was decreased at week six compared to baseline in the aspirin arm, but not in the placebo. The change in the Shannon index between week 0 and week 6 was statistically significant in the aspirin arm but not in the placebo arm (Shannon index change of −0.206 in the aspirin arm and −0.108 in the placebo arm, *p* = 0.01 and 0.19, respectively) (Supplemental Table S1, Supplemental Figure S1). The ANOSIM analysis indicated that the change in microbiome composition was significant in the aspirin arm but not in the placebo arm (R = 0.08 for pre vs. post aspirin, *p* < 0.001 vs. R = 0.0004 for pre vs. post-placebo, *p* = 0.42) (Supplemental Table S2), but we did not find an association between the composition of oral microbiome samples and aspirin intervention at week 6 using PERMANOVA (Supplemental Table S3) or Principal Coordinates Analysis (Supplemental Figure S2).

### 3.3. Analysis of a Priori Selected Taxa

**Figure 2:**
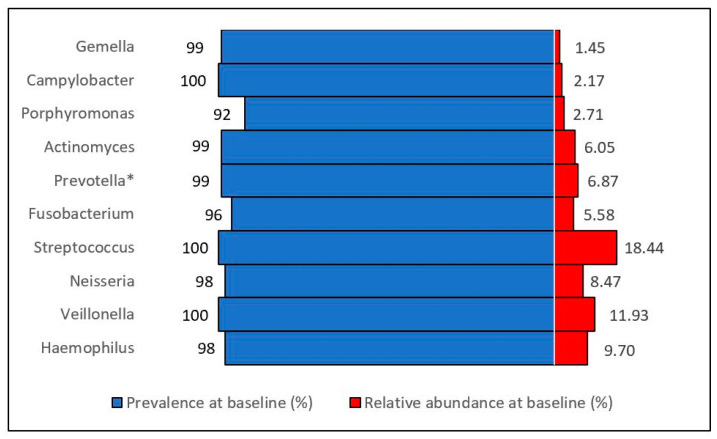
Distribution of pre-specified oral taxa (genus level) in the ASMIC trial. Prevalence represents the detection prevalence (presence vs. absence of taxa across samples, %). Abundance represents the relative abundance (i.e., the presence of a particular organism compared to all other organisms in a given sample, %). * The *Prevotella* genus level was reported as “*Prevotella_D_7*” by QIIME2 version 2023.10.

**Figure 3:**
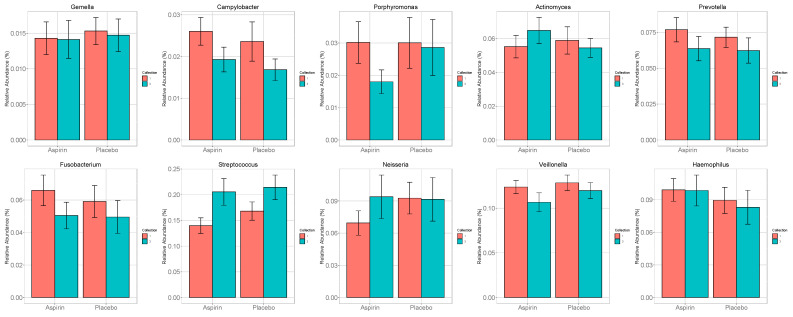
Change in relative abundance of pre-specified taxa in the aspirin and placebo groups from baseline (week 0) to post-intervention (week 6).

At baseline, the prevalence (presence/absence) for our a priori selected oral taxa ranged from 95% to 100%, but their relative abundance was low (1.4–18.4% of all oral taxa at baseline) (Figure 2) Compared to the placebo, in the aspirin group, there were more significant increases in the relative abundances of *Neisseria*, *Streptococcus*, *Actinomyces*, and *Rothia,* and more significant decreases in the relative abundance of *Prevotella*, *Veillonella*, *Fusobacterium*, and *Porphyromonas* at the genus level (Figure 3, Supplemental Table S4).

### 3.4. Analysis of Log Ratios

**Figure 4:**
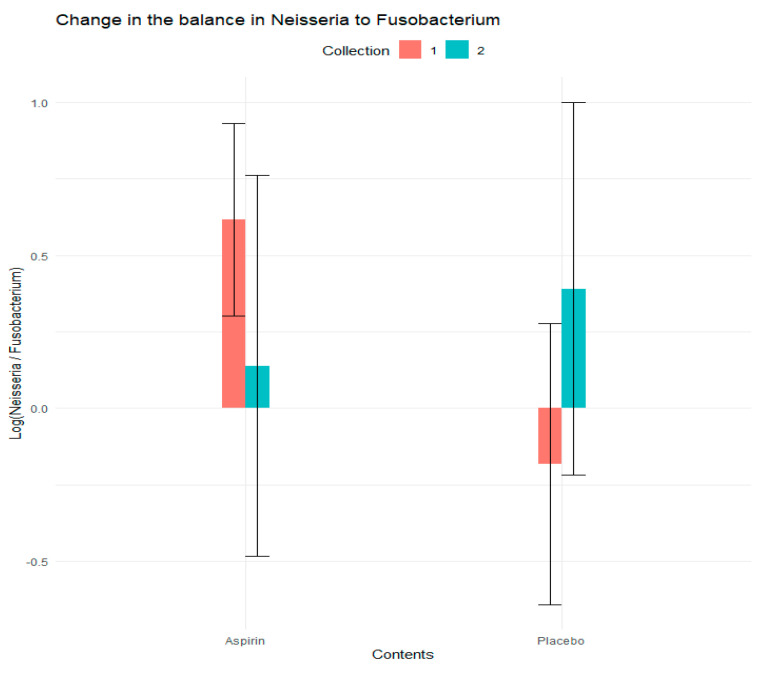
Change in the balance in Neisseria to Fusobacterium (at the family level) in the aspirin and placebo group from baseline (week 0) to post-intervention (week 6).

We assessed the abundance of log ratios (henceforth referred to as balances) and the change in the relative abundance of all a priori taxa relative to *Fusobacterium* in response to the treatment. We observed that the log-ratio of *Neisseria* to *Fusobacterium* changed more in the aspirin group than the placebo group (−1.06 vs. 0.51, *p* = 0.04), suggesting that in addition to the observed change in relative abundance for *Neisseria* and *Fusobacterium*, the ratio of these organisms may serve as a marker of microbiome changes in response to aspirin modulation (Table 2 and Figure 4).

### 3.5. Differential Analysis Based on DESeq2

Our differential analysis found no association between the fold change in relative abundance of oral taxa and aspirin intervention at week 6 (Supplemental Tables S5 and S6).

## 4. Discussion

This pilot longitudinal study of aspirin and oral microbiome in 50 healthy individuals found that the temporal changes in the relative abundance of CRC-associated bacterial taxa differed between the aspirin and placebo groups. In the aspirin group, there were greater increases in the relative abundances of oral *Neisseria*, *Streptococcus*, *Actinomyces*, and *Rothia,* and greater decreases in the relative abundance of oral *Prevotella*, *Veillonella*, *Fusobacterium*, and *Porphyromonas* compared to the placebo group. More specifically, the decline in the log ratio value in the aspirin group suggests that the relative abundances of *Fusobacterium* changed more in the aspirin group compared to the placebo group, in agreement with our hypothesis that aspirin may modulate the abundance of specific bacteria which may be of importance in the development of CRC, as *Fusobacterium* is a known pro-inflammatory taxon. However, the associations with specific bacterial groups were rather modest, and we were unable to test direct associations between these oral taxa and periodontitis or CRC as this was outside the scope of this pilot study of healthy participants.

In addition, our findings of a decrease in the relative abundances of *Prevotella*, *Veillonella*, *Fusobacterium*, and *Porphyromonas* in the aspirin group are consistent with their understood role in the CRC in previous studies, which showed that these taxa are more abundant in the oral microbiome [7,9,24] and gut microbiome [4,5,7,9,24,54,56,57,58] of CRC cases compared to cancer-free individuals. In the present study, we could not directly evaluate the association between these taxa and CRC, given that our study enrolled healthy participants. However, these findings align with the role of these bacteria in CRC and the previously reported protective association between aspirin treatment and CRC risk [22,59,60]. In particular, the present study also found a decrease in the relative abundance of *Fusobacterium* and *Porphyromonas* in the aspirin arm, and the pro-inflammatory properties of these bacteria were reported in animal models [31]. This is consistent with a recent study that reported aspirin’s direct inhibitory effect on *Fusobacterium* growth in vitro [38,39]. Our findings of an increase in the relative abundance of *Neisseria*, *Streptococcus*, and *Actinomyces* are also consistent with their understood role in the oral microbiome, as these taxa are often associated with oral health or considered commensals in the oral cavity. A recent study of oral taxa and inflammation also reported that a shift to the oral health-associated taxon *Corynebacterium* from the disease-associated pro-inflammatory taxon *Treponema* (based on their log ratio) was associated with poor periodontal health and cardiometabolic markers early in disease pathogenesis in both subgingival plaque and saliva [15], which is consistent with our observed shift to the health associated taxon *Neisseria* from the disease associated taxon *Fusobacterium* in our current study.

Although oral taxa are primarily involved in upper GI malignancies and periodontal, respiratory, and cardiovascular diseases [7,61,62], these bacteria also have a role in local and systemic chronic inflammation. Previous studies have found that circulating levels of inflammatory markers, such as C-reactive protein (CRP) [63] and phospholipase A2(PLA2) [64] levels, are associated with specific members of the oral microbiota. Our results align with these findings and suggest aspirin may influence the oral microbiome in a direction consistent with decreasing inflammation.

In addition to the potential role of pro-inflammatory oral taxa in promoting local and systemic inflammatory responses, we also hypothesized that oral taxa could be translocated to the gut microenvironment, as oral taxa such as *Fusobacterium* and *Porphyromonas* are known to work synergistically and possess several virulence factors that may promote bacterial survival along the GI tract through the development of biofilms [13,24,25,31]. Although the exact mechanisms behind the translocation of oral bacteria to the gut microbiome remain to be understood, a “driver-passenger” model has been proposed [4], and the biological traits of several oral taxa of interest, including their ability to form biofilms [58] and serve as adhesion factors [24,64], make it plausible for oral bacteria to survive the transfer from the oral cavity to the colon and adhere to the colonic mucosa. Further, the oral microbiome has been associated with various cancers along the GI tract [14], including head and neck cancer [65], esophageal cancer [5], lung cancer [66], pancreatic cancer [5], and CRC [7,9,24]. Taken together, these findings suggest that the oral microbiome may be a risk factor for CRC and should be further investigated along with the gut microbiome to understand the microbiome’s role in CRC.

Few studies have examined the association between oral bacterial taxa and inflammation in the context of CRC risk [7,9,14,67]. One study of 99 persons with CRC found that *Haemophilus*, *Parvimonas*, *Prevotella*, *Alloprevotella*, *Lachnoanaerobaculum*, *Neisseria, and Streptococcus* were less abundant in individuals with CRC than in healthy individuals [9]. These findings agree with our *Neisseria* and *Streptococcus* findings but contrast with the findings for *Prevotella*. We note that findings for *Prevotella* are inconsistent across studies [7,67]. A nested case-control study of 231 cases and 432 controls in the Southern Community Cohort Study found that *Treponema denticola* and *Prevotella intermedia* were associated with increased CRC risk [67], which agrees with our findings for *Prevotella.* Another study conducted among 190 participants in a population-based case controls study found that CRC history was associated with increased abundance of the oral genus *Rothia* [60] in age- and batch-adjusted models. This contradicts our findings of a greater relative abundance of *Rothia* in the aspirin group post-intervention. These differences could be explained by taxa’s high genomic diversity and functionality at the species and strain taxonomic level. The inconsistent associations in various studies may also reflect the complex nature of the microbiome in which the detected changes in specific bacterial taxa may reflect changes in the whole system that we cannot assess. However, our exploratory analysis using balances was designed to account for changes in the relative abundance of unobserved oral taxa, and the findings from these exploratory analyses confirmed the findings of our primary analysis.

Aspirin intervention was unrelated to α-diversity, β-diversity, or overall differential abundance, suggesting that aspirin treatment has a limited effect on variation among core taxa. Aspirin could influence bacterial taxa via either systemic or local mechanisms. A systemic mechanism of aspirin might involve changes to the tumor microenvironment [38], the inactivation of cyclooxygenase enzymes, the subsequent suppression of prostaglandins, and the production of anti-inflammatory lipoxins [36], leading to the clearance of inflammatory bacteria by macrophages and other immune cells. A local mechanism may be explained by the formation of salicylic acid from aspirin in the liver and its permeation into human GI tissue [37,68], where salicylic acid may come in contact with enteric bacteria after ingestion. Aspirin-induced changes in the oral microbiome would likely influence the GI tract via systemic inflammation [69].

The absence of an association between oral microbiota and aspirin at the community level was unexpected. However, given the proposed mechanisms of action of aspirin on the microbiome via suppressing systemic inflammation, it is plausible that the effect of aspirin on oral bacterial taxa would be modest and not reflected by the changes in overall composition. The current literature assumes that gut dysbiosis is most important in developing an inflammatory phenotype, and our results indicate that aspirin treatment was associated with a decrease in the relative abundance of several a priori selected oral taxa known to be pro-inflammatory. This highlights the importance of studying microbial communities in various sites along the digestive tract for a more comprehensive understanding of dysbiotic states and their impact on inflammation. 

Our study has some important limitations, including limited data on potential confounders, a small sample size, and a modest duration. Although we did not collect detailed information about oral hygiene practices, dental disease history, diet, or smoking, which are likely critical determinants of the oral microbiome, including a placebo arm and randomized allocations allowed us to limit the influence of unmeasured confounders. In addition, given our healthy sample, we could not evaluate specific taxa that are overrepresented in CRC patients, but rarely observed in healthy people. However, our main objective was to evaluate the influence of aspirin on 14 lingual bacteria predisposing to inflammation and CRC, and we found that aspirin changed several bacterial taxa in a direction consistent with reduced CRC risk. Finally, the results presented here are likely influenced by the methods employed to collect and measure oral microbes. Variability occurs even within the oral cavity, partly due to spatial variations in the availability of oxygen [70,71]. The oral samples collected as tongue swabs in this study are likely to overrepresent microbes present on the surface of the oral cavity, as well as saliva, and are less likely to include microbes from dental plaques [71], which may have different roles in dysbiotic oral conditions [71]. Nonetheless, oral sample collection was conducted by our trained researchers in a manner that provided some consistency. Another limitation was that 16S rRNA sequencing targeted only the V4 hypervariable region, rather than the V3–V4 hypervariable regions, which is recommended for unbiased coverage of important microbial taxa in the oral microbiome [10]. Although we were able to detect all our a priori selected lingual taxa with relative abundances ranging from 1.45% to 18.44% at baseline, these findings should be interpreted with caution and validated in studies using either 16S sequencing of the V3–V4 region or metagenomic sequencing. Additional methods, such as 16S sequencing of the V3–V4 region or metagenomic sequencing, may provide a more comprehensive understanding of more detailed insights into the abundance and function of important taxa in various oral ecological niches.

## 5. Conclusions

In conclusion, this double-blind, randomized, placebo-controlled pilot trial suggests that aspirin induces changes in several lingual bacteria previously shown to be associated with CRC or inflammation. We identified changes in the relative abundance of a priori selected taxa that agree with an inverse association between aspirin use and CRC risk. We observed a more significant increase in commensal and health-associated taxa and a more significant decrease in pro-inflammatory taxa in the aspirin group compared to the placebo group. However, these preliminary findings are limited by the lack of data on dental hygiene, diet, and smoking status and should be validated in larger studies using metagenomic sequencing. Our double-blind randomized study may inform the design of future, more extensive studies, including oral examinations, and the use of metagenomic sequencing for in-depth microbiome analyses.

## Figures and Tables

**Table 1:** ASMIC study descriptive statistics.

Characteristics	Aspirin Group	Placebo Group	*p*-Value
n (%)	30 (60.0%)	20 (40.0%)	
Age, Mean (SD) y	62.2 (5.1)	61.2 (5.2)	0.56
Sex, Female%	23 (76.7%)	9 (45.0%)	0.02
BMI, Mean (SD), kg/m^2^	27.3 (4.3)	28.2 (4.8)	0.50
Baseline urinary PGE-M (adjusted for creatinine, mg/dL)	11.82 (13.59)	12.94 (7.39)	0.73
Change in urinary PGE-M at 6 weeks (adjusted for creatinine, mg/dL)	−5.31 (0.96)	0.86 (1.18)	<0.01

**Table 2:** Effect of aspirin treatment on the change over time in the balance of *Neisseria* relative to *Fusobacterium* in the aspirin and placebo group.

Balance Ratio	Predictor	Average Change in Balance in the Aspiring Group	Average Change in Balance in the Placebo Group	Estimate	Std. Error	Z Value	Pr (>|z|)
Neisseria to Fusobacterium Ratio	Placebo (vs. Aspirin)	−1.060	0.508	−0.80	0.36	−2.20	0.03
	Collection 2 (vs. Collection 1)			−0.48	0.32	−1.47	0.14
	Intervention X Collection Interaction			1.05	0.51	2.05	0.04

## Data Availability

The data that support the findings of this study are available upon request from the corresponding author (A.E.P.). The data are not publicly available due to the regulations on clinical trial data.

## Supplementary Materials

The following supporting information can be downloaded at: https://www.mdpi.com/article/10.3390/microorganisms12081609/s1, Supplemental Figure S1. Pairwise test for the change in Shannon alpha-diversity within the aspirin and placebo arm. Supplemental Figure S2. Principal Coordinates Analysis (PCOA) plot for the Beta diversity in the oral microbiome samples at in the aspirin and placebo treatment arms at week 6. Supplemental Table S1: Association between aspirin treatment and post-intervention alpha diversity (week 6). Supplemental Table S2: Analysis of similarities (ANOSIM) between the Aspirin and placebo arms at baseline and post-intervention. Supplemental Table S3: Association between treatment and post-intervention beta diversity (week 6). Supplemental Table S4: Effect of aspirin treatment on the change over time in abundance of pre-specified bacterial taxa at the genus level using linear mixed effect models. Supplemental Table S5: Differential abundance (genus level agglomeration) between the Aspirin and Placebo groups post-intervention (crude model). Supplemental Table S6: Differential abundance (genus level agglomeration) between the Aspirin and Placebo groups post-intervention (adjusted model).
