# Supplementary material for: The Effects of Aspirin Intervention on Inflammation-Associated Lingual Bacteria: A Pilot Study from a Randomized Clinical Trial
Source: Microorganisms. 2024 Aug 7;12(8):1609. doi: 10.3390/microorganisms12081609 (PMC11357305; doi:10.3390/microorganisms12081609)
Supplement: Supplementary file 1 [file microorganisms-12-01609-s001.zip › Supplemental Tables and Figures.pdf]

Supplemental Tables and Figures

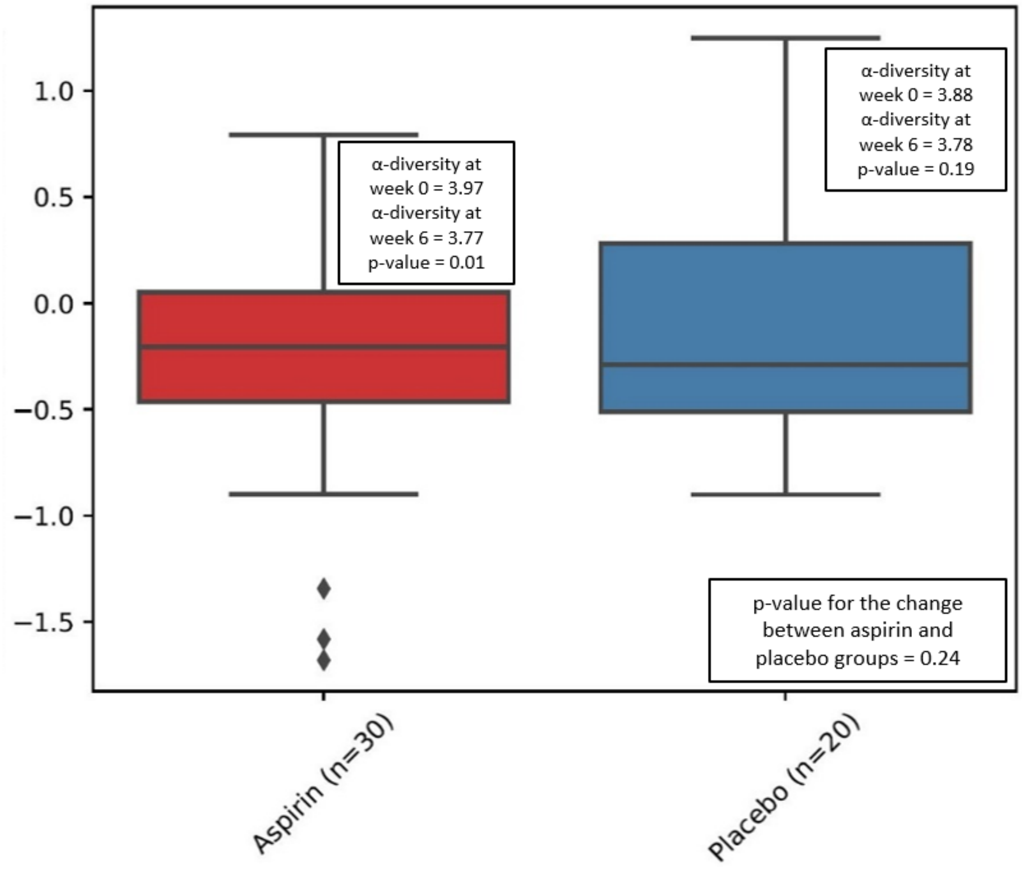

Supplemental Figure S1. Pairwise test for the change in Shannon alpha-diversity within the aspirin and placebo arm.

*The Wilcoxon signed rank test was used to test for significance within each arm, and the Kruskal-Willis test was used to test for overall significance.

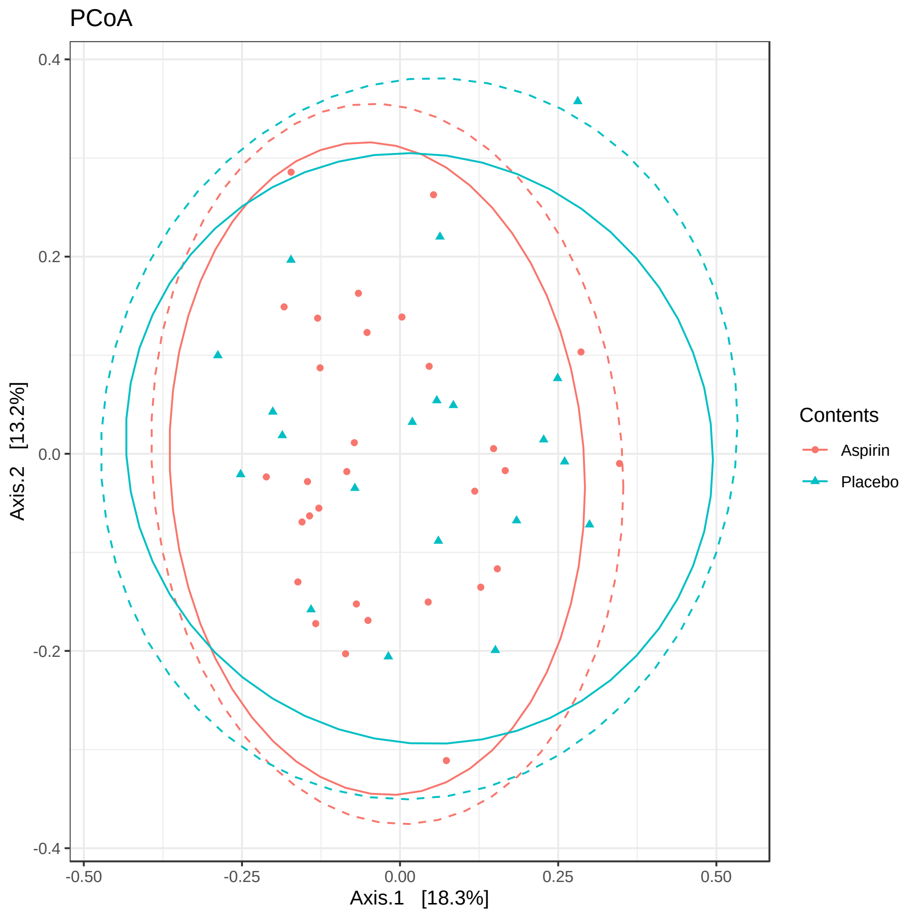

Supplemental Figure S2. Principal Coordinates Analysis (PCOA) plot for the Beta diversity in the oral microbiome samples at in the aspirin and placebo treatment arms at week 6.

Supplemental Table S1: Association between aspirin treatment and post-intervention alpha diversity (week 6)*

			Adjusted Models*			
Alpha Diversity Measure	Change in the Aspirin group	Change in the Placebo group	Estimate	Std. Error	t-value	Pr(>|t|)
Shannon	-0.206	-0.108	-0.018	0.133	-0.133	0.89

* **Adjusted models post intervention.** The adjusted model was adjusted for, age, gender, and BMI.

Supplemental Table S2: Analysis of similarities (ANOSIM) between the Aspirin and placebo arms at baseline and post intervention

Anosim Comparison Group	R-value	*P-value
Aspirin arm at week 0 vs. Aspirin arm at week 6	0.078	<0.001
Aspirin arm at week 0 vs placebo arm at week 0	0.057	0.10
Aspirin arm at week 0 vs. placebo arm at week 6	0.149	<0.05
Aspirin arm at week 6 vs. placebo arm at week 0	0.103	<0.05
Aspirin arm at week 6 vs. placebo arm at week 6	0.042	0.14
Placebo arm at week 0 vs. placebo arm at week 6	-0.0003	0.42

*The p-value is based on the non-parametric test of the rank order of dissimilarity values between the groups.

Supplemental Table S3: Association between treatment and post-intervention beta diversity (week 6)

Time Point	Df	SumsOfSqs	MeanSqs	F.Model	R2	Pr(>F)**
Post intervention crude model	1	0.14	0.14	0.97	0.02	0.41
Post intervention adjusted model*	1	0.15	0.15	1.04	0.02	0.32

* The model was adjusted for, age, gender, and BMI

** The presented p-value is for the PERMANOVA F test.

Supplemental Table S4: Effect of aspirin treatment on the change over-time in abundance of pre-specified bacterial taxa at the genus level using linear mixed effect models.

Taxa	Predictor	Average change in relative abundance pre to post intervention (% Change, Aspirin Arm)	Average change in relative abundance pre to post intervention (% Change, Placebo Arm)	Crude Models			Multivariate Models		
				Estimate	Std. Error	Pr(>|z|)	Estimate	Std. Error	Pr(>|z|)
Streptococcus	Placebo (vs. Aspirin)	6.498	4.704	0.219	0.190	0.248	0.238	0.202	0.237
	Collection 3 (vs. Collection 1)			0.473	0.009	<2e-16	0.429	0.009	<0.001

	Intervention * Collection Interaction			-0.152	0.013	<2e-16	-0.109	0.013	<0.001
Veillonella	Placebo (vs. Aspirin)	-1.653	-0.995	0.014	0.105	0.894	0.016	0.112	0.889
	Collection 3 (vs. Collection 1)			0.004	0.013	0.782	0.003	0.013	0.825
	Intervention * Collection Interaction			-0.095	0.020	0.000	-0.094	0.020	<0.001
Haemophilus	Placebo (vs. Aspirin)	-0.018	-0.556	-0.157	0.199	0.431	-0.033	0.192	0.862
	Collection 3 (vs. Collection 1)			0.068	0.015	0.000	0.075	0.015	<0.001
	Intervention * Collection Interaction			0.046	0.023	0.050	0.039	0.024	0.101
Neisseria	Placebo (vs. Aspirin)	2.409	-0.033	0.370	0.333	0.268	0.267	0.341	0.433
	Collection 3 (vs. Collection 1)			0.461	0.016	<2e-16	0.461	0.016	<0.001

	Intervention * Collection Interaction			-0.294	0.024	<2e-16	-0.294	0.024	<0.001
Prevotella_7	Placebo (vs. Aspirin)	-1.191	-1.135	-0.116	0.170	0.494	-0.093	0.181	0.607
	Collection 3 (vs. Collection 1)			0.035	0.020	0.087	0.050	0.021	0.015
	Intervention * Collection Interaction			-0.074	0.032	0.020	-0.089	0.032	<0.001
Actinomyces	Placebo (vs. Aspirin)	0.981	-0.554	0.175	0.177	0.323	0.131	0.186	0.483
	Collection 3 (vs. Collection 1)			0.241	0.023	<2e-16	0.244	0.023	<0.001
	Intervention * Collection Interaction			-0.468	0.036	<2e-16	-0.471	0.036	<0.001
Fusobacterium	Placebo (vs. Aspirin)	-1.580	-0.854	-0.133	0.280	0.636	-0.155	0.300	0.605
	Collection 3 (vs. Collection 1)			-0.210	0.023	<2e-16	-0.203	0.023	<0.001

	Intervention * Collection Interaction			0.167	0.036	0.000	0.160	0.036	<0.001
Porphyromonas	Placebo (vs. Aspirin)	-1.252	-0.193	0.010	0.312	0.974	0.156	0.321	0.627
	Collection 3 (vs. Collection 1)			-0.414	0.050	<2e-16	-0.413	0.050	<0.001
	Intervention * Collection Interaction			0.403	0.065	0.000	0.403	0.065	<0.001
Campylobacter	Placebo (vs. Aspirin)	0.019	0.017	-0.099	0.183	0.588	-0.161	0.176	0.361
	Collection 3 (vs. Collection 1)			-0.191	0.059	0.001	-0.190	0.059	0.001
	Intervention * Collection Interaction			-0.197	0.099	0.046	-0.197	0.099	0.047
Gemella	Placebo (vs. Aspirin)	0.014	0.015	0.129	0.191	0.499	0.160	0.204	0.434
	Collection 3 (vs. Collection 1)			0.126	0.084	0.134	0.129	0.084	0.125

Intervention * Collection Interaction	-0.048	0.134	0.718	-0.052	0.134	0.700

*Multivariate models were adjusted for age, gender, and BMI

Supplemental Table S5: Differential abundance (Genus level agglomeration) between the Aspirin and Placebo groups post intervention (crude model)

Taxa	baseMean	log2FoldChange*	lfcSE	stat	pvalue	padj
Bacteroides	9.24	-4.72	1.76	-2.68	0.01	0.43
Johnsonella	5.46	5.18	2.28	2.28	0.02	0.67
Haemophilus	2679.93	-0.73	0.56	-1.32	0.19	1.00
Veillonella	2772.40	0.30	0.29	1.05	0.30	1.00
Neisseria	2331.23	-0.30	0.50	-0.60	0.55	1.00
Streptococcus	6313.31	-0.19	0.39	-0.48	0.64	1.00
Fusobacterium	1094.39	-0.09	0.45	-0.21	0.84	1.00
Prevotella_7	1467.83	0.22	0.31	0.70	0.48	1.00
Granulicatella	1046.33	0.04	0.36	0.11	0.91	1.00
Actinomyces	1467.06	-0.09	0.30	-0.29	0.77	1.00
Rothia	2806.87	-0.37	0.49	-0.75	0.45	1.00
Porphyromonas	511.51	0.43	0.65	0.67	0.51	1.00
Campylobacter	398.74	-0.03	0.30	-0.09	0.93	1.00
Leptotrichia	709.42	-0.52	0.40	-1.30	0.19	1.00
Gemella	376.00	-0.16	0.43	-0.39	0.70	1.00
Atopobium	172.64	0.53	0.44	1.20	0.23	1.00
Megasphaera	129.60	0.24	0.55	0.43	0.67	1.00
Moryella	59.24	-0.08	1.00	-0.08	0.94	1.00
Prevotella_6	132.37	0.29	0.46	0.62	0.53	1.00

*All log fold change values for the post intervention (week 6) DESeq2 analysis are for the abundance in the placebo group vs. the aspirin group.

Supplemental Table S6: Differential abundance (Genus level agglomeration) between the Aspirin and Placebo groups post-intervention (adjusted model)

Taxa	baseMean	log2FoldChange**	lfcSE	stat	pvalue	padj
Bacteroides	9.24	-6.57	2.09	-3.14	0.00	0.10
Johnsonella	11.44	5.98	2.42	2.47	0.01	0.40
Haemophilus	2679.93	-0.22	0.60	-0.38	0.71	0.99
Veillonella	2772.40	0.36	0.29	1.24	0.22	0.99
Neisseria	2331.23	-0.22	0.54	-0.40	0.69	0.99
Streptococcus	6313.31	-0.20	0.42	-0.48	0.63	0.99
Fusobacterium	1094.39	-0.05	0.49	-0.11	0.92	0.99
Prevotella_7	1467.83	0.12	0.32	0.37	0.71	0.99
Granulicatella	1046.33	0.06	0.38	0.14	0.89	0.99
Actinomyces	1467.06	-0.23	0.32	-0.72	0.47	0.99
Rothia	2806.87	-0.17	0.51	-0.34	0.73	0.99
Porphyromonas	511.51	0.43	0.71	0.60	0.55	0.99
Campylobacter	398.74	-0.08	0.31	-0.25	0.81	0.99
Leptotrichia	709.42	-0.61	0.41	-1.47	0.14	0.99
Gemella	376.00	0.03	0.45	0.06	0.95	0.99
Atopobium	172.64	0.36	0.47	0.77	0.44	0.99
Megasphaera	129.60	0.15	0.60	0.25	0.80	0.99
Moryella	59.24	-0.84	3.11	-0.27	0.79	0.99
Prevotella_6	132.37	0.35	0.49	0.72	0.47	0.99

* The negative binomial model was adjusted for age, gender, and BMI

**All log fold change values for the post intervention (week 6) DESeq2 analysis are for the abundance in the placebo group vs. the aspirin group.
